# Quantifying training response in cycling based on cardiovascular drift using machine learning

**DOI:** 10.3389/frai.2025.1623384

**Published:** 2025-07-04

**Authors:** Artur Barsumyan, Raman Shyla, Anton Saukkonen, Christian Soost, Jan Adriaan Graw, Rene Burchard

**Affiliations:** ^1^Faculty of Medicine, Philipps-University of Marburg, Marburg, Germany; ^2^Sports Medicine and Joint Centre, Department of Orthopedics and Trauma Surgery, Lahn-Dill-Kliniken, Dillenburg, Germany; ^3^Department of Mathematics and Systems Analysis, Aalto University, Espoo, Finland; ^4^Faculty III: Statistic and Econometrics, University of Siegen, Siegen, Germany; ^5^Department of Anaesthesiology and Intensive Care Medicine, Ulm University Hospital, Ulm, Germany; ^6^Department of Orthopedics and Traumatology, University Hospital of Giessen and Marburg, Marburg, Germany

**Keywords:** cardiovascular drift, machine learning, cycling, aerobic fitness, Gaussian process

## Abstract

**Purpose:**

The most important parameter influencing performance in endurance sports is aerobic fitness, the quality of the cardiovascular system for efficient oxygen supply of working muscles to produce mechanical work. Each individual athlete responds differently to training. However, for coaches it is not always easy to see improvement, accumulated fatigue, or overreaching. In the new era of technology, we propose an experimental method using machine learning (ML) to measure response quantified as aerobic fitness level based on cardiovascular drift and aerobic decoupling data.

**Methods:**

Twenty well-trained athletes in cycling-based sports performed monthly aerobic fitness tests over five months, riding at 75% of their functional threshold power for 60 min. Based on aerobic decoupling (power-to-heart rate ratio) and cardiovascular drift of each test ride, a prediction model was created using ML (Logistic regression, Variational Gaussian Process models and k-nearest neighbors algorithm) that indicated whether or not an athlete was responding to the training. Athletes were spitted as responders (i.e., those showing improvements in cardiovascular drift and aerobic decoupling) or non-responders.

**Results:**

Cardiovascular drift and aerobic decoupling demonstrated a significant strong linear correlation. All ML models achieved good predictive performance in classifying athletes as responders or non-responders, with cross-validation accuracy ranging from 0.87 to 0.9. Average predictive accuracy of 0.86 was for k-nearest neighbors, 0.91 for logistic regression, 0.93 for Variational Gaussian Process model. The Variational Gaussian Process model achieved the highest classification for training response.

**Conclusion:**

Cardiovascular drift and aerobic decoupling are reliable indicators of response to training stimulus. ML is a promising tool for monitoring training response in endurance sports, offering early and sensitive insights into fitness adaptations or fatigue that can support more personalized training decisions for coaches and athletes.

## 1 Introduction

Aerobic fitness is fundamental for success in endurance sports, reflecting the capacity of the cardiovascular and metabolic systems to sustain prolonged exercise (MacInnis and Gibala, 2017). High aerobic fitness, often indexed by maximal oxygen uptake (VO₂max), enables athletes to maintain output with greater efficiency (Jones, 2023). During steady-state endurance exercise, a phenomenon known as cardiovascular (CV) drift is commonly observed: over time, heart rate gradually rises despite a constant workload (Souissi et al., 2021). This drift is accompanied by a decline in stroke volume and arterial pressure. This is attributed to physiological adjustments such as increased skin blood flow (redirecting blood from central circulation) and elevated sympathetic drive together prompting a compensatory heart rate increase (Ferri Marini et al., 2022). In well-trained athletes, cardiovascular drift tends to be attenuated; a smaller rise in heart rate for a given power output is often interpreted as a sign of robust aerobic endurance (Hellsten and Nyberg, 2015; Ferri Marini et al., 2022). Conversely, excessive drift can signify fatigue or underdeveloped aerobic base, as the cardiovascular system struggles to maintain output over time (Lajoie et al., 2000). Recent evidence supports this practical insight, showing that athletes who exhibit minimal cardiovascular drift or aerobic decoupling during prolonged exercise perform better in endurance events (Smyth et al., 2022). A large study in marathon runners found that those with the lowest heart rate–pace decoupling maintained higher speeds and achieved faster finish times compared to those with a greater drift. This relationship has led to the concept of using heart rate drift as an indicator of endurance “durability,” where a stable heart rate relative to output denotes superior aerobic fitness (Hunter et al., 2025).

Modern advancements in sports technology have transformed how athletes and coaches monitor training and detect cardiovascular drift in real time (Seçkin et al., 2023; Assalve et al., 2024). Wearable devices such as chest-strap heart rate monitors, GPS watches, and cycling power meters continuously track internal load (heart rate), external load (speed or power), or mix of load (power-to-heart rate ratio), providing detailed data on an athlete’s physiological responses (Gao et al., 2018). These tools enable objective assessment of heart rate behavior during workouts and races. For example, wireless heart rate monitoring has been used by cyclists for years to gauge training intensity and detect early signs of CV-drift or overtraining (Migliaccio et al., 2024). Similarly, power meters measure output (e.g., watts in cycling or running) with high precision, allowing the calculation of heart rate–to–power ratios over time (Andriolo et al., 2024; De Leeuw et al., 2025). The integration of heart rate and power data has given rise to metrics like power-heart rate decoupling, which are now accessible to athletes outside of laboratory settings. The continuous stream of data from these devices helps in quantifying an athlete’s training response: coaches can observe how an athlete’s heart rate trends relative to a constant pace or power and adjust training accordingly (Muggeridge et al., 2021). The proliferation of wearable sensors and performance tracking has led to an explosion of physiological data, from daily training sessions to entire season logs. These big data hold valuable information about an athlete’s fitness and fatigue status, but its volume and complexity pose analytical challenges (Halson, 2014). Traditional statistical methods often struggle to interpret the nonlinear, multivariate relationships inherent in such physiological datasets. By contrast, modern machine learning (ML) techniques excel at extracting hidden patterns from complex data and can handle large-scale, continuous records more effectively (Boudry et al., 2024). ML algorithms have already shown promise in endurance sports science. For example, they have been used to predict key fitness indicators like VO₂max from routine training data, providing practical alternatives to exhaustive laboratory tests (Beltrame et al., 2017). The strength of ML lies in its ability to model interactions between numerous variables (heart rate, power, duration, environmental factors, etc.) and to learn from longitudinal data of individual athletes (Reis et al., 2024; Beato et al., 2025). This makes it a powerful approach for interpreting cardiovascular drift in context. Furthermore, ML enables real-time analysis: recent studies have demonstrated that ML models fed with wearable sensor data can continuously estimate an athlete’s physiological state and even deliver personalized exercise feedback based on individual responses (Fang et al., 2024; Zhu, 2025). Such capabilities highlight why applying ML to heart rate drift data is a logical next step in advancing endurance training science. Modern wearable devices have made cardiovascular drift and aerobic decoupling (Power: heart rate) metrics accessible to athletes, but most monitoring systems remain descriptive and retrospective in nature. Current tools often lack the capacity to process and interpret high-volume, high-frequency data in a way that supports real-time, personalized decision-making. This highlights a critical gap that machine learning techniques are well-positioned to address.

Despite the widespread availability of wearable sensors, there remains a lack of predictive tools that can utilize continuous heart rate and power data to assess an athlete’s aerobic training response in a meaningful and individualized way. This study seeks to address this gap by applying machine learning to cardiovascular drift data as a practical indicator of training effectiveness in well-trained athletes.

The present study aimed to investigate the use of ML algorithms to analyse cardiovascular drift and aerobic decoupling as a real-time, personalized indicator of aerobic fitness in endurance athletes. We tried to determine whether ML-driven models of cardiovascular drift (during sustained, steady-state exercise) can reliably track or predict positive response to training. In other words, athletes with higher aerobic fitness (as measured by traditional benchmarks) will exhibit distinctive cardiovascular drift profiles that our ML models can identify.

## 2 Materials and methods

### 2.1 Participants and data collection

Twenty-one well trained healthy individuals (21 males, age 31 ± 3 years; training > 6 h per^.^week were recruited for this study). All athletes had at least 4 years of structured endurance training experience, primarily in road and gravel cycling. Participants were recruited via regional cycling clubs and personal contacts, resulting in a convenience sample of well-trained, competitive cyclists. While this approach may introduce some selection bias, the study was designed as an exploratory investigation within a performance-homogeneous population. All subjects were screened and confirmed free from chronic illnesses or conditions known to influence cardiovascular function. One participant was excluded due to acute cholecystitis during the study period, leaving a final sample size of 20 athletes.

This study was conducted in accordance with the Declaration of Helsinki (2013) and the ethics committee of the Philipps University of Marburg approved the study (24–327 RS). Informed consent was obtained from all participants after they had been informed verbally and in writing about the experimental protocol. The study was not formally registered. The data are available on reasoned request to the corresponding author.

#### 2.1.1 Experimental setup

Each participant performed a standardized cycling test monthly for a period of 5 months. Tests were integrated into the participants’ regular training schedules as a controlled training session. Each test consisted of a 10-min incremental warm-up followed by a 60-min steady-state effort at 75% of their current functional threshold power (FTP). FTP was verified and updated monthly using the cycling analytics software WKO 5 (build 590, Peaksware LLC, Lafayette, CO, USA), ensuring the intensity remained consistent with the athlete’s current fitness level. Participants were not blinded to their power output during testing, as visual feedback was necessary to maintain the prescribed target intensity of 75% FTP.

To minimize confounding factors affecting cardiovascular drift, such as environmental conditions, hydration status, circadian rhythms, and nutritional influences, a standardized testing protocol was strictly enforced. All tests occurred at the same time of day in a controlled indoor environment with adequate ventilation and fan cooling directed towards the participants’ torso. Fluid intake was standardized at 500 mL h^−1^ of water or electrolyte solution. Participants refrained from food consumption during the tests and were instructed to consume a standardised meal 2 h before the test, eliminate caffeine 4 h prior, and avoid alcohol and strenuous exercise 24 h preceding the test. Between tests, participants followed their habitual coach-directed training plans. Training content and load were not standardized, as the goal was to evaluate physiological responses under realistic, valid training conditions.

Tests were performed on participants’ personal racing bicycles mounted on electromagnetically braked, direct-drive cycling trainers (Kickr v5, Wahoo Fitness, Atlanta, USA or Tacx, Wassenaar, Netherlands). Trainers were calibrated according to manufacturer guidelines to ensure reliable performance data. Power output was recorded using either Garmin Rally (Garmin Ltd., Olathe, Kansas, USA) or Favero Assioma (Favero Electronics, Treviso, Italy) power meters, paired to Garmin Edge devices (Edge 520 or Edge 1,040, Garmin Ltd., Olathe, Kansas, USA). Participants completed a zero-offset calibration prior to each session as per manufacturer instructions. Heart rate data were recorded continuously throughout each test using a heart rate chest strap (Garmin HRM-Pro, Garmin Ltd., Olathe, Kansas, USA) coupled with portable head units.

### 2.2 Data analysis and preprocessing

Data collection was performed, analysed and visually inspected for errors by two independent researchers using commercially available cycling software TrainingPeaks (version 9.3.0, Peaksware LLC, Lafayette, CO, USA) and WKO 5. Sessions exhibiting physiological artifacts or technical errors—such as sudden spikes or drops in power not accompanied by corresponding changes in cadence or heart rate, prolonged zero-output segments during active testing, or loss of signal due to device malfunction—were excluded from analysis. No data imputation was performed, and only complete, clean sessions were included for model training and evaluation. The variables extracted for each test included FTP (watt), heart rate (bpm), average power output (watt), cardiovascular drift, aerobic decoupling.

Cardiovascular drift was calculated using following Equation 1:


(1) $$\text{Cardiac Drift}\;(\%)=\frac{\mathrm{HR}(\text{Second Half})-\mathrm{HR}(\text{First Half})}{\mathrm{HR}\;(\text{First Half})}x100$$

And aerobic decoupling Equation 2:


(2) $$\mathrm{Pw}:\mathrm{Hr}\;\text{Decoupling}\;(\%)\frac{\mathrm{Pw}:\mathrm{Hr}\;(\text{First Half})-\mathrm{Pw}:\mathrm{Hr}\;(\text{Second Half})}{\mathrm{Pw}:\mathrm{Hr}\;(\text{First Half})}x100$$

### 2.3 Machine learning development

Since the goal of this study is to train ML models and maximize their predictive accuracy on out-of-distribution data, we have chosen two non-parametric ML models as the main candidates. In addition to that, we have included one simpler model candidate which we use as a benchmark. The simpler model is the logistic regression, which predicts the probability of a binary outcome using the logistic function (Zaidi and Al Luhayb, 2023). The candidate models are K-nearest neighbors (KNN) algorithm, which uses non-parametric techniques to classify a sample based on the majority vote of its KNN (Shi, 2020) and Variational Gaussian Process (VGP), which uses a non-parametric variational inference integration technique to approximate the posterior distribution over functions and performs probabilistic classification (Rasmussen and Williams, 2006). All three models were implemented to identify the most suitable and accurate predictive model for classifying athletes’ training responses based on cardiovascular drift.

We selected the three models—logistic regression, kNN, and VGP—based on complementary modeling strengths suited to the characteristics of our dataset. kNN and VGP were chosen because both are non-parametric models known to perform well in small data regimes. Their flexibility allows them to model complex relationships without requiring strong parametric assumptions, which are often difficult to justify or verify in real-world sports science data. Logistic regression was included as a widely used and interpretable baseline model. Its simplicity makes it a useful reference point against which to compare the performance of more flexible models like kNN and VGP. Together, these models provide a balanced perspective: from a classical linear baseline to non-parametric methods with differing complexity and generalization behavior.

The ML models were structured as follows: logistic regression utilized a standard linear classifier with L2 regularization, which is necessary to improve stability and generalization quality for predictive purposes. The KNN algorithm was optimized using Euclidean distance. Optimal k value was selected with line search, using stratified 10-fold cross-validation within each training set, repeated across multiple random seeds to ensure robustness (see Model Validation chapter). The best-performing k was then used in the final reporting. Gaussian Process classification employed the Matern52 kernel function and was optimized by minimizing Kullback–Leibler divergence with gradient-type method. Prior to model training, all input features were standardized using z-score normalization (zero mean, unit variance).

In addition, we placed strong emphasis on the dataset development for our experiment. Specifically, we optimized the models using three forms of the dataset: the original, the difference-transformed dataset as described in Results and the transformation using the Principal Component Analysis (PCA) technique (Gewers et al., 2021). We discussed the caveats of each representation in detail in the Results chapter, highlighting how each transformation impacts model performance and interpretability.

The study used Python 3.12.3., with libraries such as scikit-learn, GPflow, numpy, and TensorFlow (Pedregosa et al., 2011; Abadi et al., 2015).

### 2.4 Performance evaluation

We assessed athletes’ responses to training by comparing the results of two consecutive tests. Given that each of the 20 athletes completed one standardized test monthly over a period of 5 months, a total of 80 paired comparisons were obtained (four comparisons per athlete). Specifically, each athlete’s test results at the beginning of 1 month were compared with the subsequent test results 1 month later. A positive training response was defined as an improvement in cardiovascular drift and aerobic decoupling metrics, whereas the absence of such improvement indicated no response to the preceding training. This month-to-month comparison allowed for a detailed assessment of individual training adaptations over the course of the study.

For further analysis, responses were coded in a binary format: a positive response was recorded as “1” and no response as “0.” This binarization enabled structured data handling and facilitated modeling of training adaptations. A “responder” was defined as an athlete who demonstrated a physiologically meaningful improvement between two consecutive tests, reflected by a combined decrease in both cardiovascular drift (i.e., a smaller rise in heart rate relative to power over time) and aerobic decoupling. These changes indicate enhanced cardiovascular stability and efficiency, consistent with improved aerobic fitness or durability. Conversely, a “non-responder” was defined as an athlete who exhibited no meaningful change or a worsening in one or both metrics compared to the prior test. This includes scenarios where cardiovascular drift or aerobic decoupling remained stable or increased, suggesting stagnation, accumulated fatigue, or a lack of adaptation to the preceding training load.

For the purposes of modeling these responses, we treated each of the 80 comparisons as approximately independent observations, given the presence of random factors influencing individuals’ performance improvements. Furthermore, the models employed—logistic regression with L2 regularization, KNN, and Gaussian processes—either do not rely on parametric assumptions (KNN and VGP), or, in the case of logistic regression, remain valid when used strictly for predictive purposes.

To evaluate model accuracy, we used precision, recall, and F1 score, which offer a balanced view of classification quality. To evaluate model performance, we employed a stratified 10-fold cross-validation process (StratifiedKFold), which helps preserve the percentage of samples for each class in each fold, ensuring that each fold is representative of the overall distribution of the data. For each fold, accuracy scores were recorded, and these were averaged to obtain a reliable estimate of the model’s performance across different training and validation splits. To further assess the model’s robustness, the model was retrained on the entire training dataset using the optimized hyperparameters and evaluated on the unseen test set. The final performance of the model was assessed based on its accuracy in predicting the test set, with the results of both the cross-validation and the unseen test set being used to compare the models’ predictive power (Wilimitis and Walsh, 2023).

## 3 Results

### 3.1 Model validation

The hyperparameters of each model were tuned, and the generalizability of each model was tested using a custom cross-validation procedure. This procedure involved splitting the data into multiple subsets while incorporating two sets of random seeds to ensure that the results were unbiased and reproducible. First, the data were split into a training and a testing set using a randomized seed for the split. A second layer of randomization was applied during the k-fold cross-validation (k-FCV) process to further enhance the robustness of the evaluation and reduce the influence of any random initialization on the final results.

Specifically, two sets of random seeds were used: one for the initial train-test split (denoted as seeds_o) and another for the stratified k-fold cross-validation within each training set (denoted as seeds_i). The use of different seed sets ensured that both, the train-test splitting and the subsequent k-fold validation process were independent, reducing any potential bias that may arise from a specific choice of seed. The seed values for both, the splits and the folds were shuffled to eliminate any patterns that might skew the model evaluation, allowing the performance metrics to reflect the generalizability of the model rather than artifacts from data splitting.

The overall procedure began by performing a randomized train-test split with 80% of the data used for training and 20% for testing. When PCA was utilized, the training data were first transformed using PCA to reduce dimensionality before applying it to the test data.

### 3.2 Exploratory data analysis and feature engineering

**Figure 1:**
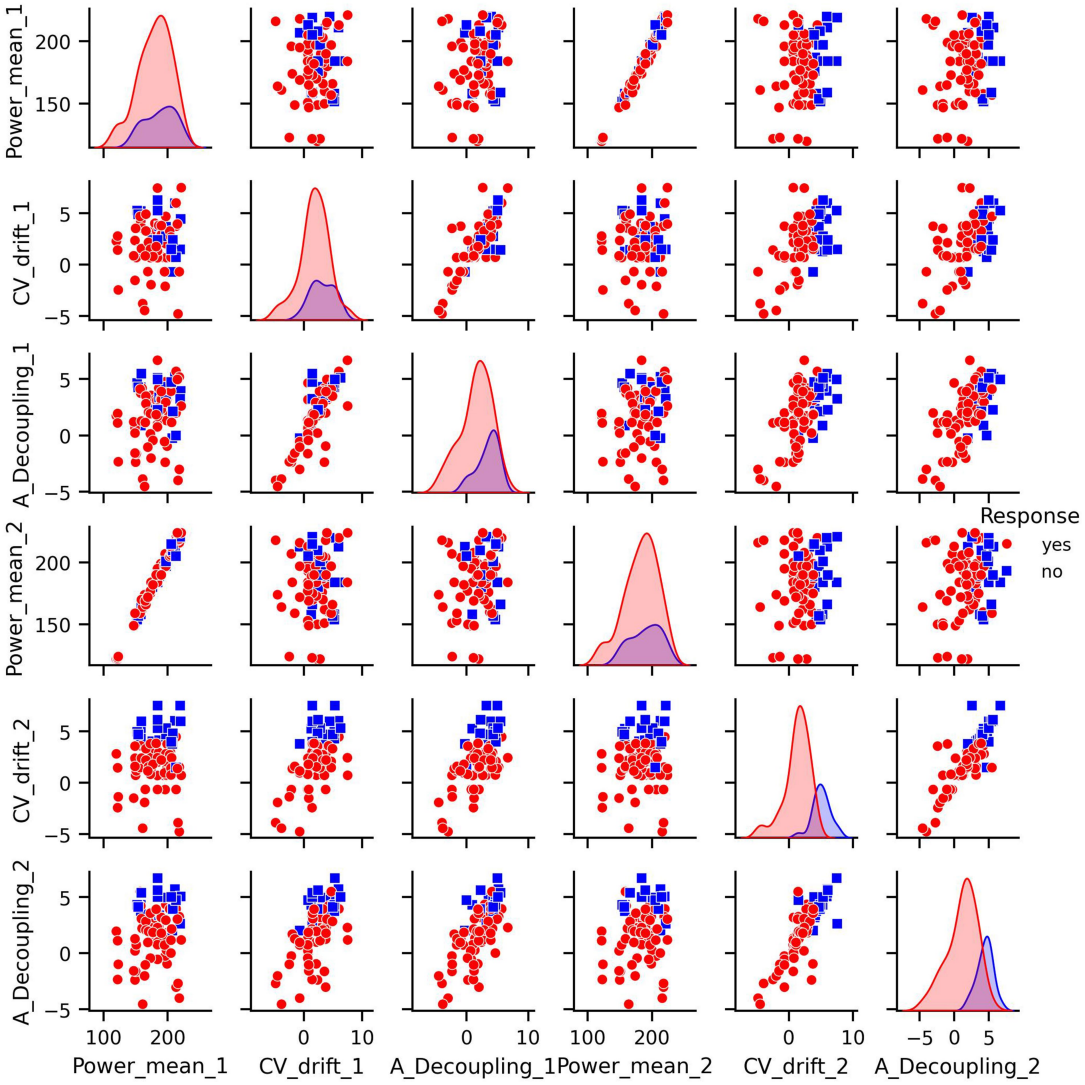
Pair plots of covariates in original dataset.

**Figure 2:**
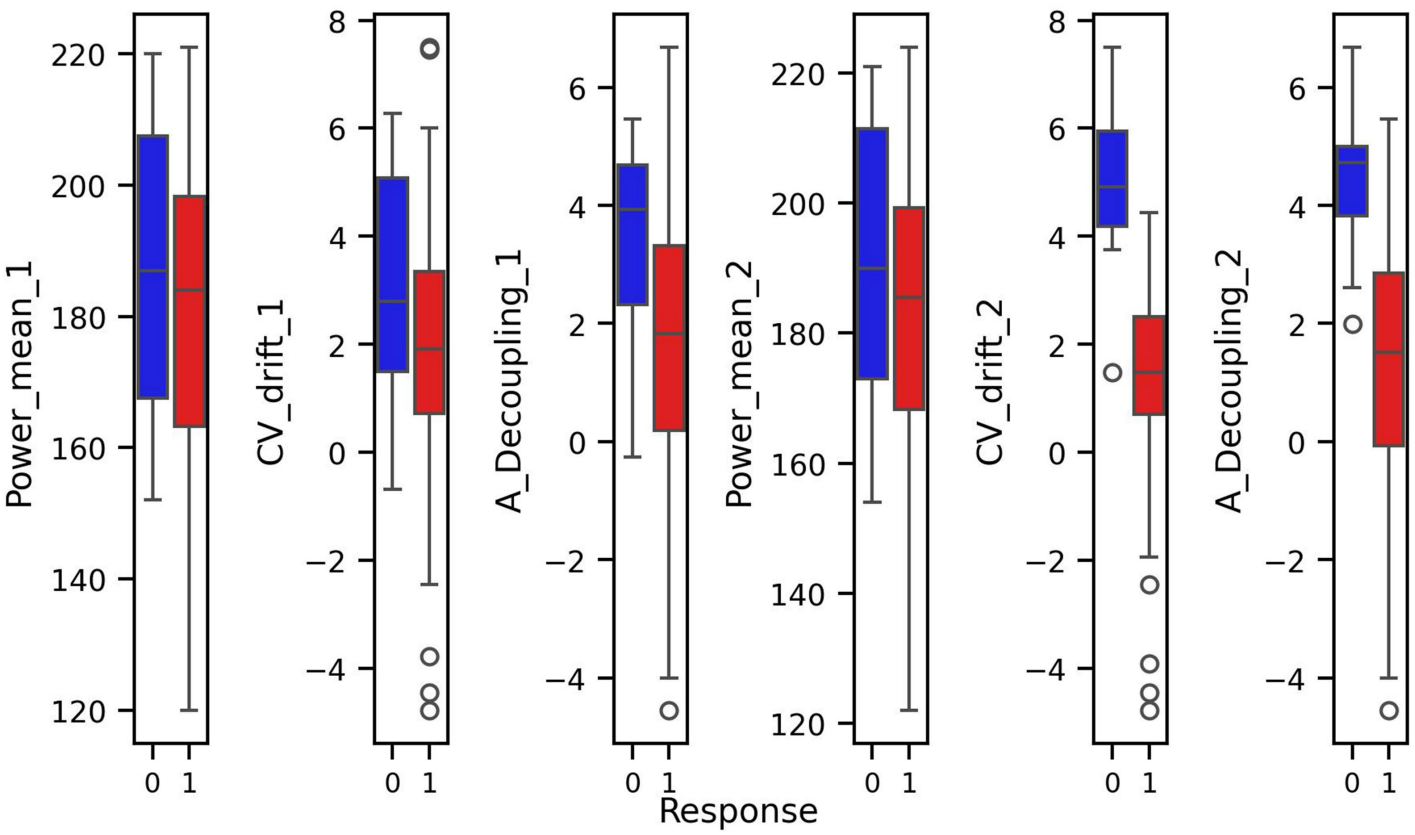
Box plots of covariates with respect to response and non-response groups.

Prior to modeling, we conducted exploratory data analysis to better understand the structure of the features and their relationship to the response variable. Visualizations including pair plots and box plots (Figures 1, 2) highlighted strong linear dependencies between several raw features—e.g., CV-drift and aerobic decoupling across test intervals—as well as indications that certain metrics may be more predictive than others.

For every figure in this manuscript, Power_mean_1 represents the mean power output (in watts) from the initial test, CV_drift_1 indicates the cardiovascular drift (%) observed during the initial test, and A_Decoupling_1 refers to aerobic decoupling (%) from the same session. Similarly, Power_mean_2, CV_drift_2, and A_Decoupling_2 correspond to the same metrics collected during the comparison test. Each point in the scatter plots is color-coded according to the response outcome: red circles represent responders (yes), and blue squares indicate non-responders (no).

In particular, the box plots revealed that the most pronounced differences between the response groups were found in cardiovascular drift_2 and aerobic decoupling_2, which suggest that these features may serve as key indicators of an athlete’s adaptation to training (Figure 2).

**Figure 3:**
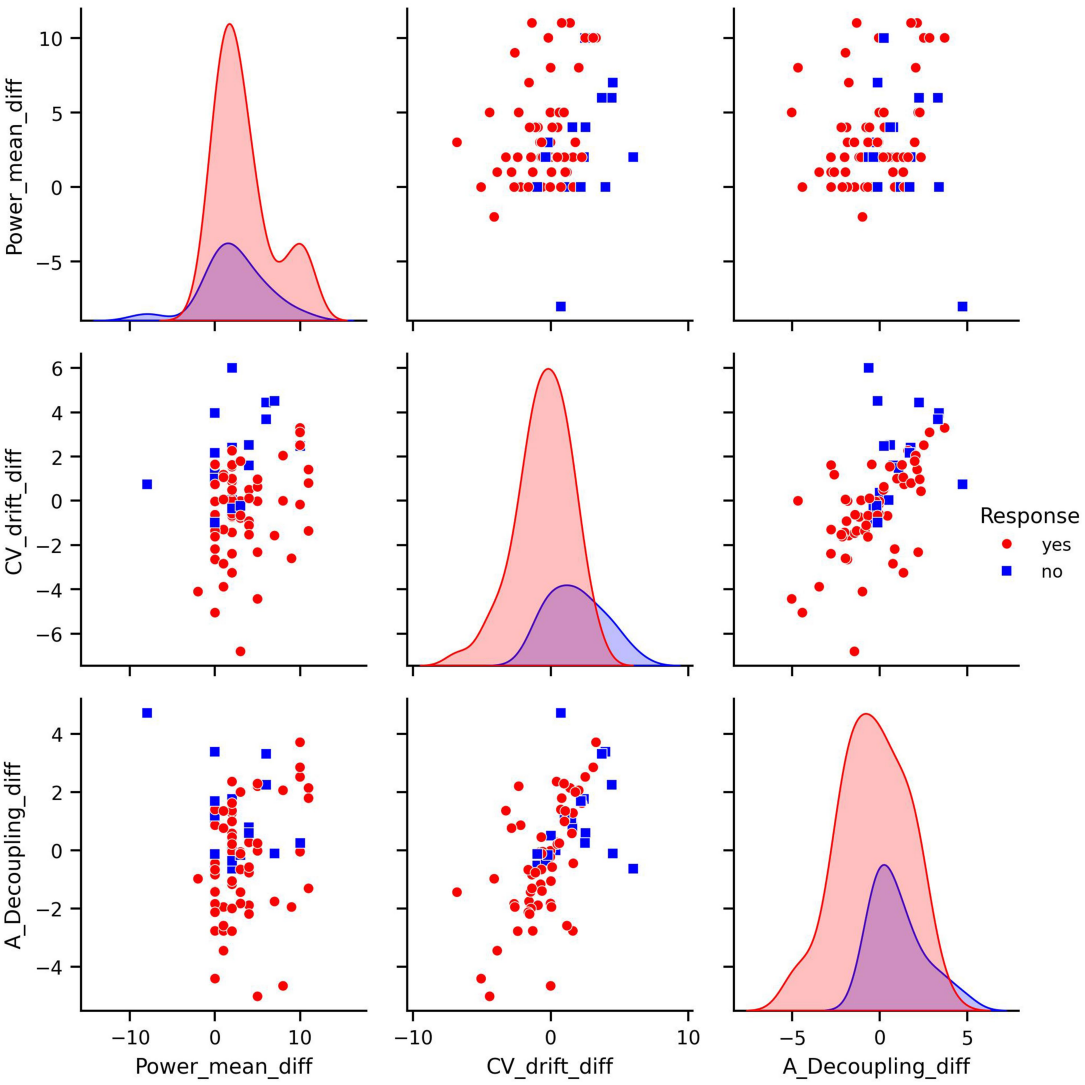
Pair plots of covariates in difference transformed dataset.

**Figure 4:**
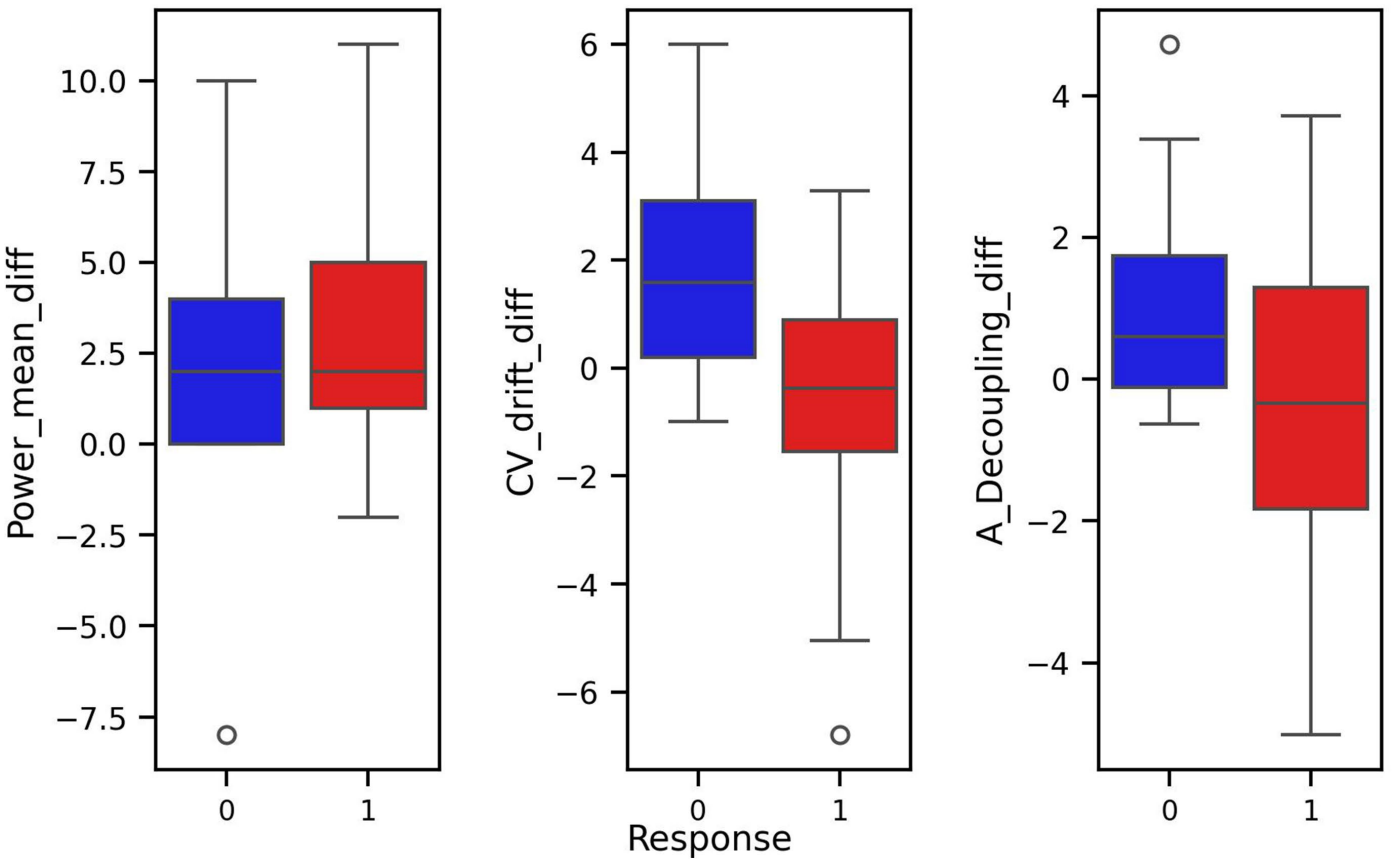
Box plots of covariates of difference transformed dataset with respect to response and non-response groups.

To reduce redundancy and potentially improve model performance, we transformed the dataset by computing differences between consecutive measurements. Such transformation allows to represent features as relative changes, achieved during the training period, rather than absolute values recorded on specific test intervals (Figures 3, 4). This transformation aligns well with training theory, where improvements are typically assessed as trends over time rather than isolated snapshots. Semantically, a large negative difference in CV-drift between consecutive tests indicates its decrease over time. This can be interpreted as a positive physiological response to training. In particular, this pattern reflects improved cardiovascular efficiency. Such a change is something that an experienced coach might recognize intuitively, and therefore, it is reasonable to assume that ML model should also be able to detect and leverage this signal effectively. As a result, while some correlations remained, this feature engineering reduced multicollinearity and aligned better with the biological interpretation of a training response as a change over time.

The pair plot of the differences demonstrates that the linear dependence between CV-drift and aerobic decoupling has been reduced, though it is still present (Figure 3). The differences between the means of the response groups have shrunk, but it remains notable that they are most prominent in CV-drift and aerobic decoupling (Figure 4).

**Figure 5:**
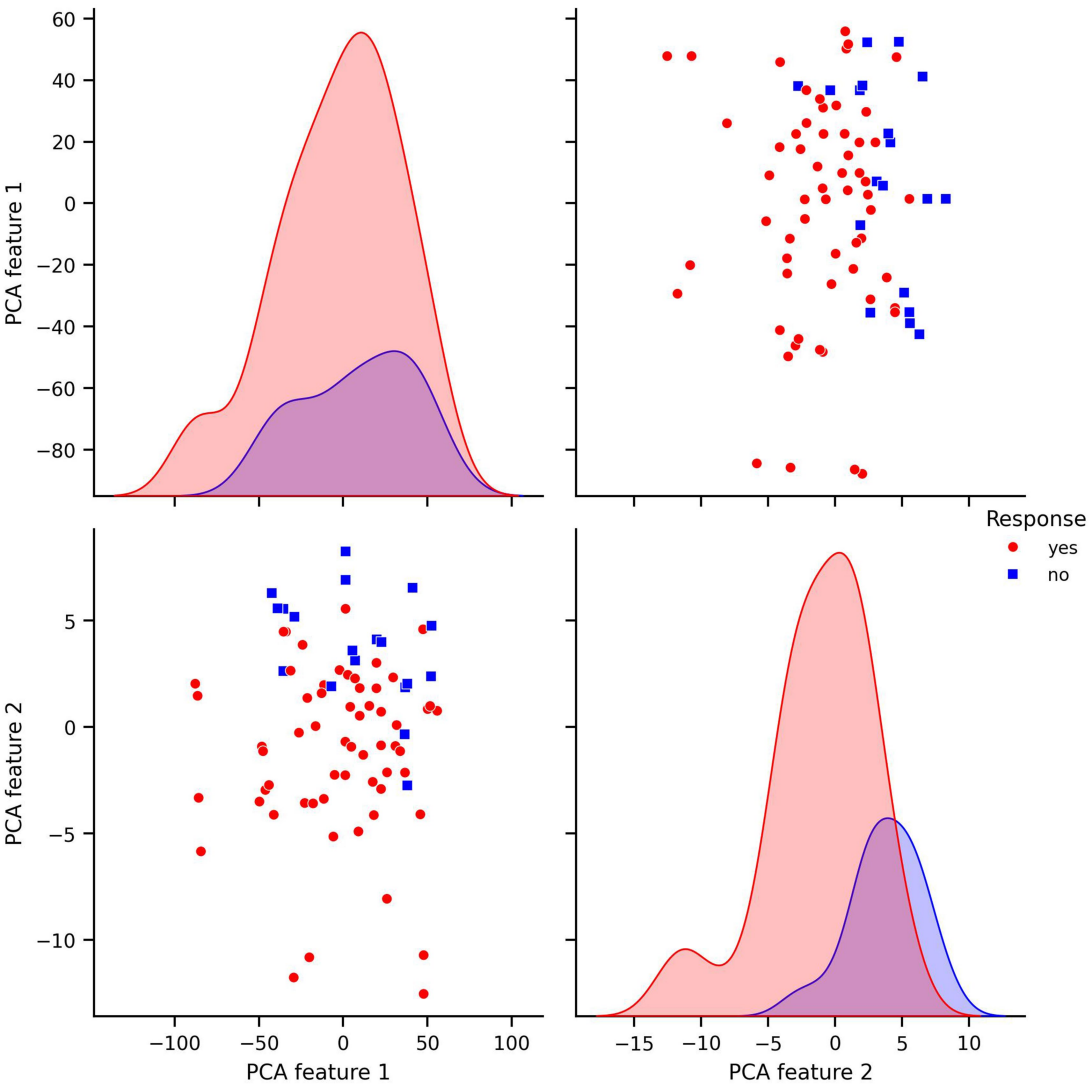
Pair plots of covariates in PCA transformed dataset.

**Figure 6:**
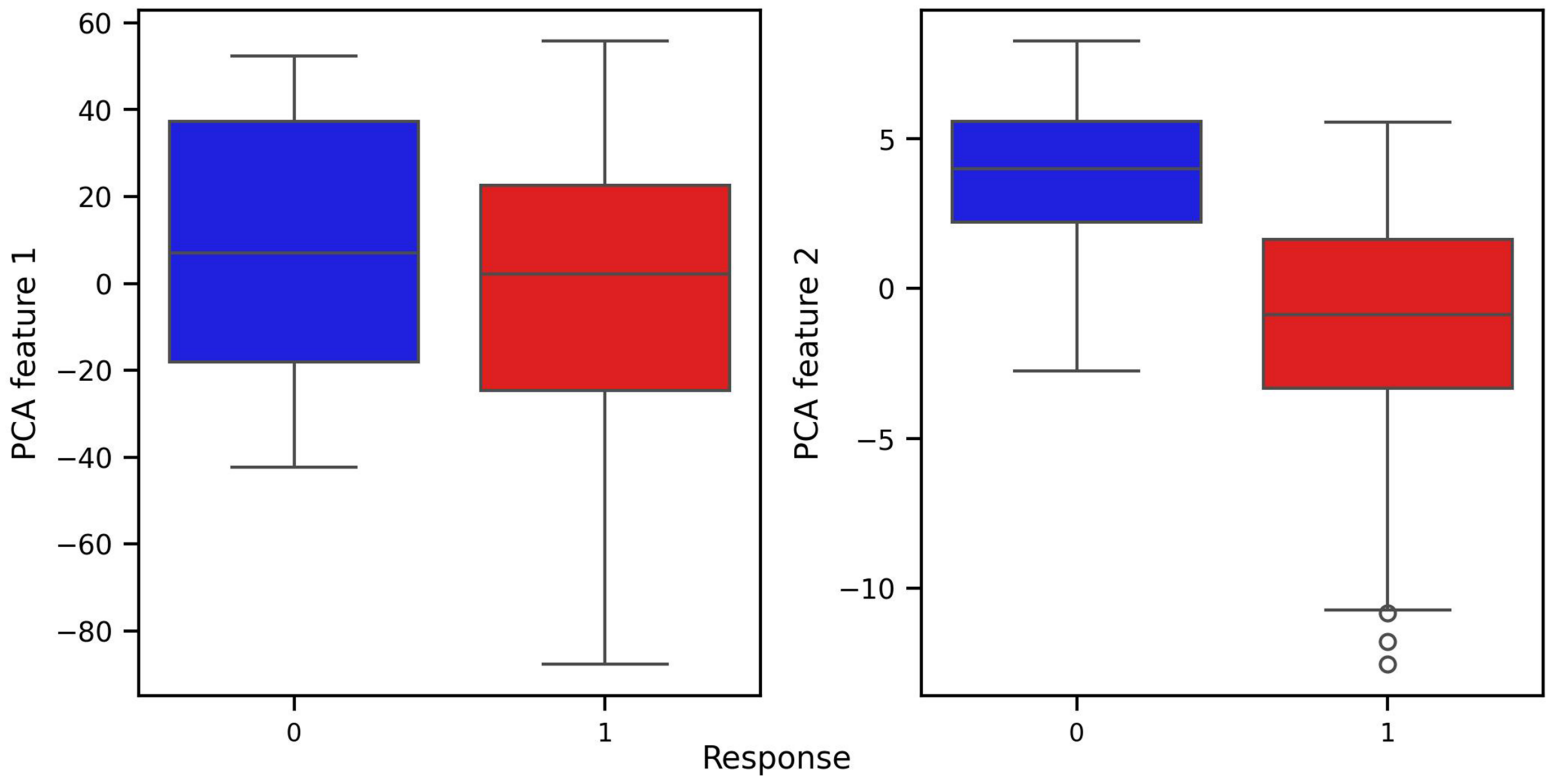
Box plots of covariates of PCA transformed dataset with respect to response and non-response groups.

Finally, another feature transformation was applied to the original dataset using the dimensionality reduction technique PCA and visualized in the corresponding pair and box plots for the PCA-transformed features (Figures 5, 6). PCA was used to reduce complexity in the modeling process and to only use covariates that correlate highly with the main components in order to rule out possible distortions caused by irrelevant covariates. At the same time, this approach increases the robustness of the overall results.

From Table 1 we can see how contribution of the features from the original dataset is reflected in the transformation under PCA.

**Table 1:** Contribution of the features under PCA transformation.

Features	PCA feature 1	PCA feature 2
Power mean 1	0.703214	0.082183
Cardiovascular drift 1	0.006465	0.486538
A decoupling 1	0.014372	0.515771
Power mean 2	0.710670	−0.109335
Cardiovascular drift 2	0.007638	0.0493126
A decoupling 2	0.011509	0.485160

The Table 1 reveals that most of the variance in PCA feature 1 is explained by the power means from tests 1 and 2, while most of the variance in PCA feature 2 is carried from cardiovascular drift and A decoupling. This separation of variance aligns well with physiological interpretations, where power output reflects to overall performance capacity, while drift and decoupling metrics reflect internal response and fatigue. Both classes of metrics are key elements in interpreting training responses.

### 3.3 Performance analysis and comparative analysis

After training and evaluating the Logistic Regression, KNN, and VGP models, we compared their performance metrics side by side, along with their computational efficiency. All three models demonstrated strong predictive accuracy in classifying training response categories. The reported outcomes reflect the highest values achieved across multiple runs, representing the best-case performance for each configuration. However, there were notable differences in computational cost. Gaussian Process models, including VGPs, are flexible and robust in small data regimes, but are also computationally intensive. In particular training scales as 𝑂(n^3^) due to the matrix inversion involved in computing the posterior over functions. This might become a limiting factor within some applications. In contrast, Logistic Regression scales much more efficiently, with training complexity of 𝑂(nd) where n is the number of samples and d is the number of features. Furthermore, KNN, takes the 𝑂(knd) cost during training, where k is the number of neighbours. These theoretical asymptotic scaling rules were confirmed during practical experiments.

Our analysis focused on evaluating the predictive performance of different models using three distinct feature representations: the original full feature set, a manually crafted difference-based transformation, and a PCA-transformed dataset. The models tested included Logistic Regression, KNN, and VGP.

The best performance across all models was achieved using the original full feature set (Table 2). Specifically, the Logistic Regression, KNN, and VGP models showed mean cross-validation accuracies of approximately 0.902, with test set accuracies reaching 0.906 and 0.931, respectively. Model performance was additionally evaluated using precision, recall and F1 score, providing a comprehensive assessment of classification quality, including both overall correctness and the balance between sensitivity and specificity (Table 3).

**Table 2:** Performance comparison of different models.

Model	Full		Diff		PCA	
	Mean CV accuracy	Mean test set accuracy	Mean CV accuracy	Mean test set accuracy	Mean CV accuracy	Mean test set accuracy
Logistic regression	0.902 ± 0.105	0.906 ± 0.031	0.827 ± 0.122	0.869 ± 0.044	0.836 ± 0.116	0.806 ± 0.086
KNN	0.869 ± 0.118	0.869 ± 0.052	0.770 ± 0.129	0.781 ± 0.075	0.771 ± 0.148	0.762 ± 0.092
VGP	0.902 ± 0.108	0.931 ± 0.044	0.811 ± 0.122	0.831 ± 0.049	0.833 ± 0.127	0.825 ± 0.083

CV, cross validation; ±, standard deviation.

**Table 3:** Comparison of evaluation of model performance using different methods.

Model	Full			Diff			PCA		
	Mean test set precision	Mean test set recall	Mean test set F1	Mean test set precision	Mean test set recall	Mean test set F1	Mean test set precision	Mean test set recall	Mean test set F1
Logistic regression	0.916 ± 0.038	0.967 ± 0.055	0.939 ± 0.021	0.864 ± 0.043	0.983 ± 0.033	0.919 ± 0.026	0.820 ± 0.068	0.958 ± 0.042	0.882 ± 0.050
KNN	0.863 ± 0.044	0.983 ± 0.033	0.919 ± 0.032	0.799 ± 0.052	0.950 ± 0.055	0.867 ± 0.046	0.822 ± 0.081	0.883 ± 0.085	0.848 ± 0.060
VGP	0.945 ± 0.036	0.967 ± 0.055	0.954 ± 0.030	0.836 ± 0.038	0.967 ± 0.055	0.896 ± 0.031	0.844 ± 0.077	0.950 ± 0.041	0.892 ± 0.049

±, standard deviation.

In contrast, the manual difference-based transformation, where metrics’ changes between test sessions for specific features were examined, consistently resulted in a decrease in predictive performance. For example, in the Logistic Regression model, the mean cross-validation accuracy dropped from 0.902 (using the full feature set) to 0.827 when using the difference-transformed features. Similarly, the test set accuracy decreased from 0.906 to 0.869. These results highlight that manually handcrafting features, while might seem potentially insightful and even intuitive for specific use cases, may also reduce the model’s ability to generalize effectively across different datasets. The performance drop suggests that this transformation might remove valuable information or fail to capture underlying patterns in the data.

The PCA transformation, which aims to reduce dimensionality while removing unnecessary complexity, showed reduced performance when compared to the full feature set. For instance, the mean cross-validation accuracy for Logistic Regression dropped to 0.836, and the test set accuracy decreased to 0.806. This result is expected since during dimensionality reduction some potentially useful information might get removed as well.

**Figure 7:**
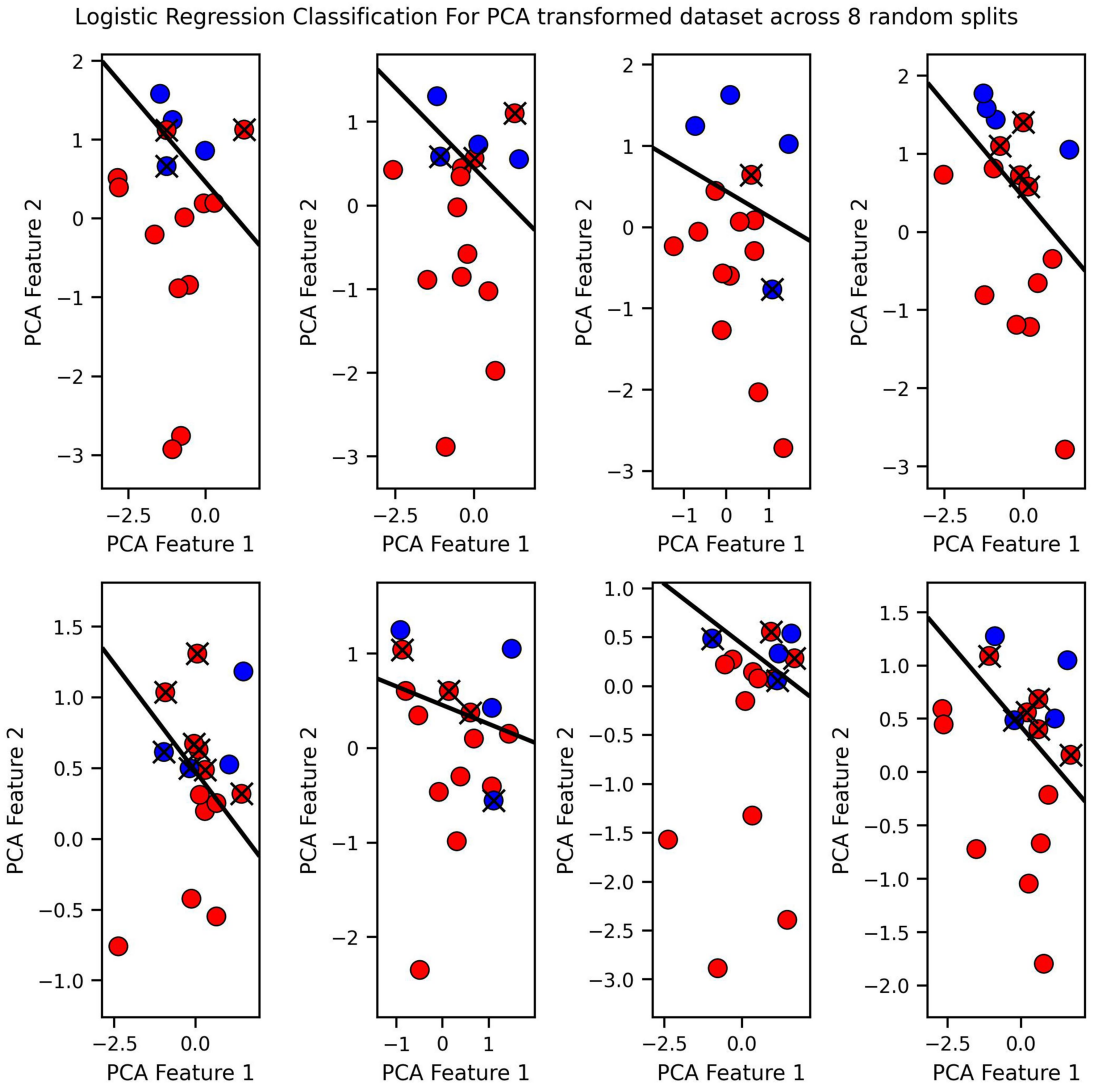
Logistic regression classification for PCA transformed dataset across 8 random splits.

**Figure 8:**
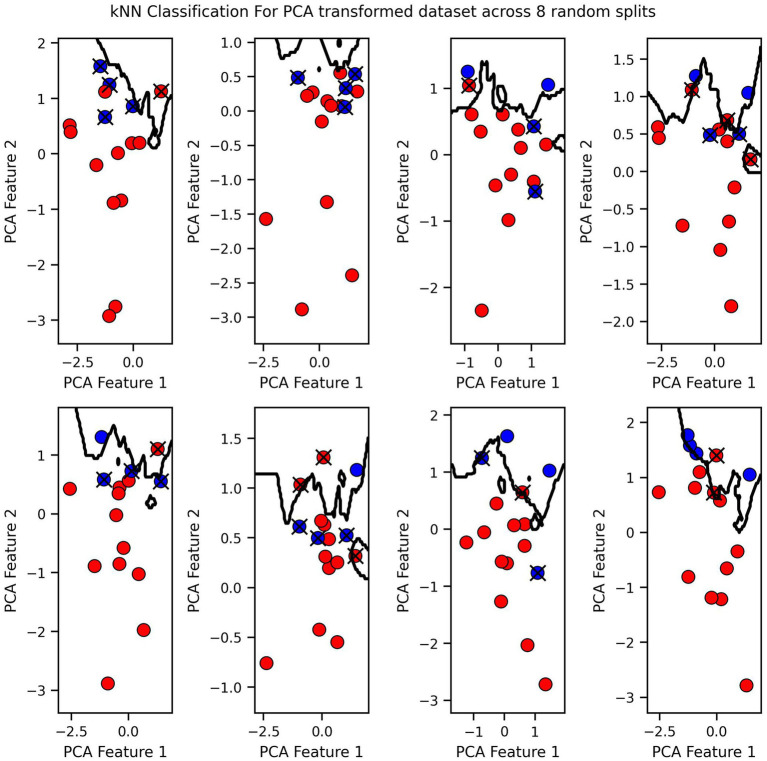
KNN Classification for PCA transformed dataset across 8 random splits.

**Figure 9:**
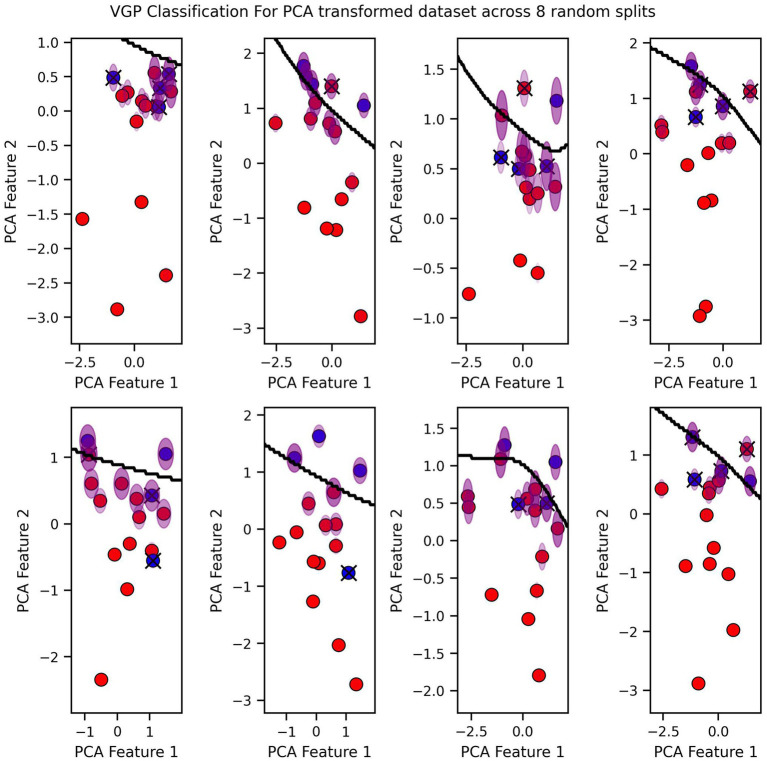
VGP Classification for PCA transformed dataset across 8 random splits.

Finally, we visualized the classification boundaries created by each of the three tested algorithms in a two-dimensional plane, after reducing the dimensionality of the original six-dimensional data using PCA (Figures 7–9).

The classification boundary produced by the VGP differs significantly from those created by KNN and Logistic Regression (Figures 7–9). As a linear classifier, Logistic Regression produced a simple, linear boundary that failed to capture the non-linear patterns in the data, leading to inaccurate predictions. In contrast, KNN generated a highly complex and irregular boundary, attempting to fit every data point, which can result in overfitting and poor generalization.

The VGP, however, created a non-linear boundary that is more flexible but not overly complex. While the model can make mistakes, it has the advantage of providing an uncertainty estimate for each prediction. The uncertainty is visualized as a purple fading circle around the point, with the radius of the circle representing the level of uncertainty with larger circles indicating higher uncertainty. This feature allows the VGP not only to make predictions but also to convey confidence in those predictions, offering a more nuanced understanding of the model’s performance. The interpretability of VGP is supported by its probabilistic nature: rather than providing solely a point estimate it also gives an uncertainty score, which can be interpreted as the model’s confidence about its prediction.

## 4 Discussion

This study aimed to investigate the response to training when estimating cardiovascular drift using ML methods. We evaluated three machine learning models, Logistic Regression, KNN and VGP, for estimating cardiovascular drift. The VGP model outperformed the others, achieving the highest cross-validation and test set accuracies (up to 0.931), likely due to its ability to capture complex patterns in small, low-dimensional datasets. Results showed that a decrease in cardiovascular drift over time, as detected by the models, corresponded to positive physiological adaptations such as improved cardiovascular efficiency. The observed reductions in cardiovascular drift and aerobic decoupling may reflect improvements in endurance-specific physiological traits—particularly durability, defined as the ability to maintain physiological stability during prolonged submaximal exercise. Lower cardiovascular drift over time suggests improved stroke volume maintenance, reduced sympathetic compensation, and enhanced thermoregulatory efficiency. These findings highlight the potential of VGP-based analysis for more sensitive and individualized monitoring of fitness improvements in sports science.

Systematic reviews show interest in using ML in sports applications as a way to optimize training, estimation of physiological thresholds and potentially increase athletes performance (Krstić et al., 2023; Zignoli, 2023). The heart rate is one of the most captured physiological metrics in sports (Lundstrom et al., 2022).

One of the key strengths of ML is its ability to handle large, high-dimensional data sets. ML algorithms can uncover complex, non-linear relationships between variables that traditional statistical methods may miss (Reis et al., 2024). This ability is particularly valuable when analysing physiological data, which often involves multiple interrelated variables (Sarker, 2021).

The capacity to process real-time data from wearable sensors is another significant advantage (Fang et al., 2024). Wearable devices continuously collect high-frequency data on variables such as HR and activity metrics. ML models can analyze these data instantaneously, providing immediate feedback that enables dynamic adjustments to training plans (Fang et al., 2024). For example, Gao et al. (2018) demonstrated how real-time analysis can enhance performance monitoring.

The effectiveness of different structured training approaches for endurance is still debated. Common models, such as various training intensity distributions based on heart rate or power zones, are frequently compared. However, studies have not consistently shown that one approach leads to superior improvements in key endurance metrics like VO₂max or time trial performance (Rosenblat et al., 2025). Indeed, the meta-analysis by Rosenblat et al. (2025) using individual participant data reported no overall significant difference in VO₂max gains or time-trial performance improvements when comparing polarized versus pyramidal intensity distributions, suggesting that different well-structured training approaches guided by effective monitoring tools (like HR monitors or power meters) can stimulate significant endurance adaptations (Rosenblat et al., 2025). These advancements underscore how technology has enhanced the monitoring of cardiovascular drift and overall training load, enabling more informed adjustments to training programs on a day-to-day basis (Fang et al., 2024; Jones, 2023; Montero, 2022; Reis et al., 2024). By integrating diverse data types, ML facilitates the analysis of individual responses to exercise, accounting for the unique physiological profile of each athlete (Muniz-Santos et al., 2023).

The primary advantage of using two separate random seed sets and multiple evaluation steps is that it helps to minimize bias from specific data splits. By averaging the results across multiple splits and folds, a more reliable estimate of model generalizability can be obtained, and by using different random seeds for the train-test split and k-fold cross-validation, it is ensured that the evaluation is not overly sensitive to a particular random initialization. While these results appear promising, the presence of multicollinearity raises concerns regarding the model’s stability and generalizability, as it can lead to overfitting in real-world data regime.

When comparing the performance of the different models, it was observed that Logistic Regression consistently outperformed KNN and VGP in terms of both cross-validation and test set accuracies across all feature transformations. In particular, the full feature set consistently provided the highest accuracy for Logistic Regression and VGP, but KNN performed similarly to GP when using the full feature set. However, the difference-transformed dataset and PCA both resulted in a drop in performance across all models, with PCA showing the least degradation in accuracy. Interestingly, while KNN was more stable with the full feature set, its performance dropped significantly with the handcrafted difference features and PCA transformation. The key advantage of PCA is that it removes multicollinearity, which can enhance model robustness. Despite the reduced predictive accuracy, the absence of multicollinearity in the PCA-transformed dataset makes it a more robust representation of the data. This suggests that while PCA might not always maximize predictive performance, it could lead to more stable and generalizable models, especially in the presence of highly correlated features, which are prominent in real world scenarios.

In conclusion, while the full feature set yields the best performance, it is likely sensitive to multicollinearity, which could affect model stability. The handcrafted difference features provide a more interpretable approach but significantly reduce model performance. PCA, although leading to a decrease in accuracy, presents a more robust solution by mitigating multicollinearity, suggesting its potential for more stable models in future applications. The choice of feature representation depends on the trade-off between predictive performance and model stability, with PCA offering a more reliable option in contexts where generalizability is important. However, the high performance of the full feature set may be influenced by multicollinearity among certain features, particularly between variables such as CV-drift and aerobic decoupling.

### 4.1 Limitations

The limited cohort size would likely negatively impact the generalizability of this work. For this reason, we strongly recommend future studies considering a larger cohort. Our study included only male athletes; future research should investigate whether similar patterns and predictive accuracy apply to female athletes, who may exhibit different cardiovascular and hormonal responses to training. Future studies should focus on expanding the dataset to capture more data from a wider range of participants with different fitness level, in order to better facilitate models that require large datasets. In this study, we did not explore factors such as accumulated fatigue, overreaching, or chronic daily stress, that might explain why some athletes did not respond. Additionally, our findings stem from a specific cycling interval protocol. Physiological responses—particularly heart rate dynamics and cardiovascular drift characteristics—may differ under steady-state conditions, at other intensities or durations, or during different exercise modalities. While the current model was developed using cycling data, its conceptual framework may be transferable to other endurance sports such as running, rowing, or cross-country skiing, which share similar cardiovascular and metabolic demands. However, sport-specific validation is essential, as biomechanical and neuromuscular factors could influence the predictive accuracy of the model. Finally, potential confounding factors common in physiological studies, such as variations in environmental conditions, diet, or prior fatigue levels, might have influenced individual responses. Employing external validation datasets and investigating alternative ML architectures would substantially strengthen the conclusions regarding the use of these methods for estimating cardiovascular responses.

## 5 Conclusion

In this study, three models of ML were evaluated. The VGP model performed better than logistic regression and KNN, which was expected due to his ability to capture more complex relationships. The results of our study demonstrated the potential value of VGP in sports field research, where data is often small sample size and low dimensional. By training on large datasets, ML models can discern how cardiovascular drift correlates with aerobic capacity or fatigue under different conditions, potentially identifying early warning signs or confirming improvements that might be missed by simple summary statistics.

These findings have practical implications for coaches and athletes. By analyzing cardiovascular drift and aerobic decoupling trends over time, ML models can help detect early signs of positive adaptation or excessive fatigue—insights that might not be apparent through traditional training metrics alone. In practice, coaches could use these models to tailor training intensity, adjust recovery strategies, or individualize periodization plans based on the athlete’s physiological feedback. Ultimately, this approach could support more responsive and personalized training prescriptions, improving both performance outcomes and athlete health management.

## Data Availability

The raw data supporting the conclusions of this article will be made available by the authors, without undue reservation.
